# Using machine learning and surface reconstruction to accurately differentiate different trajectories of mood and energy dysregulation in youth

**DOI:** 10.1371/journal.pone.0180221

**Published:** 2017-07-06

**Authors:** Amelia Versace, Vinod Sharma, Michele A. Bertocci, Genna Bebko, Satish Iyengar, Amanda Dwojak, Lisa Bonar, Susan B. Perlman, Claudiu Schirda, Michael Travis, Mary Kay Gill, Vaibhav A. Diwadkar, Jeffrey L. Sunshine, Scott K. Holland, Robert A. Kowatch, Boris Birmaher, David Axelson, Thomas W. Frazier, L. Eugene Arnold, Mary A. Fristad, Eric A. Youngstrom, Sarah M. Horwitz, Robert L. Findling, Mary L. Phillips

**Affiliations:** 1 Department of Psychiatry, Western Psychiatric Institute and Clinic, University of Pittsburgh Medical Center, University of Pittsburgh, Pittsburgh, Pennsylvania, United States of America; 2 Department of Statistics, University of Pittsburgh, Pittsburgh, Pennsylvania, United States of America; 3 Department of Psychiatry and Behavioral Neuroscience, Wayne State University, Detroit, Michigan, United States of America; 4 University Hospitals Case Medical Center/Case Western Reserve University, Cleveland, Ohio, United States of America; 5 Department of Radiology, University Hospitals Case Medical Center/Case Western Reserve University, Cleveland, Ohio, United States of America; 6 Research Institute at Nationwide Children’s Hospital, Columbus, Ohio, United States of America; 7 Department of Psychiatry, Ohio State University, Columbus, Ohio, United States of America; 8 Pediatric Institute, Cleveland Clinic, Cleveland, Ohio, United States of America; 9 Department of Psychology, University of North Carolina at Chapel Hill, Chapel Hill, North Carolina, United States of America; 10 Department of Child and Adolescent Psychiatry, New York University School of Medicine, New York City, New York, United States of America; 11 Department of Psychiatry, Johns Hopkins University, Baltimore, Maryland, United States of America; Yale University School of Medicine, UNITED STATES

## Abstract

Difficulty regulating positive mood and energy is a feature that cuts across different pediatric psychiatric disorders. Yet, little is known regarding the neural mechanisms underlying different developmental trajectories of positive mood and energy regulation in youth. Recent studies indicate that machine learning techniques can help elucidate the role of neuroimaging measures in classifying individual subjects by specific symptom trajectory. Cortical thickness measures were extracted in sixty-eight anatomical regions covering the entire brain in 115 participants from the Longitudinal Assessment of Manic Symptoms (LAMS) study and 31 healthy comparison youth (12.5 y/o;-Male/Female = 15/16;-IQ = 104;-Right/Left handedness = 24/5). Using a combination of trajectories analyses, surface reconstruction, and machine learning techniques, the present study aims to identify the extent to which measures of cortical thickness can accurately distinguish youth with higher (n = 18) from those with lower (n = 34) trajectories of manic-like behaviors in a large sample of LAMS youth (n = 115; 13.6 y/o; M/F = 68/47, IQ = 100.1, R/L = 108/7). Machine learning analyses revealed that widespread *cortical thickening* in portions of the left dorsolateral prefrontal cortex, right inferior and middle temporal gyrus, bilateral precuneus, and bilateral paracentral gyri and *cortical thinning* in portions of the right dorsolateral prefrontal cortex, left ventrolateral prefrontal cortex, and right parahippocampal gyrus accurately differentiate (Area Under Curve = 0.89;p = 0.03) youth with *different* (higher vs lower) trajectories of positive mood and energy dysregulation over a period up to 5years, as measured by the Parent General Behavior Inventory-10 Item Mania Scale. Our findings suggest that specific patterns of cortical thickness may reflect transdiagnostic neural mechanisms associated with different temporal trajectories of positive mood and energy dysregulation in youth. This approach has potential to identify patterns of neural markers of future clinical course.

## Introduction

Difficulty regulating positive mood and energy is a feature not only of pediatric bipolar spectrum disorders (BPSD),[1–3] but also of other psychiatric disorders in youth, including other mood disorders,[2, 4–6] attention deficit hyperactivity disorder (ADHD)[4, 7–10] and oppositional defiant disorders (ODD).[4, 11] It is also present in youth without a psychiatric diagnosis.[12–14] Yet, little is known regarding the neural mechanisms underlying different developmental trajectories of positive mood and energy regulation over time, and how these trajectories predispose youth to specific future psychiatric disorders. Elucidating these neural mechanisms would provide objective neural markers to help identify youth most at risk of a specific future psychiatric disorder.

The Longitudinal Assessment of Manic Symptoms (LAMS) study[15, 16] is an ongoing longitudinal follow-up of youth who were aged 6–12 years upon study entry. They were recruited via nine outpatient mental health clinics and selected based on emotional and behavioral dysregulation, regardless of diagnosis. Paralleling the NIMH’s Research Domain Criteria (RDoC) initiative on transdiagnostic studies of psychiatric illness,[17–19] the LAMS study has assessed mood symptoms and related behaviors every six months over five or more years in youth with different psychiatric disorders, including BPSD, depression, anxiety, attention deficit hyperactivity disorder (ADHD), oppositional defiant disorder (ODD) or conduct disorder (CD). One of the clinical rating scales periodically administered is the 10-item Parental General Behavioral Inventory (PGBI-10M),[20] a parental report of the child’s difficulty regulating positive mood and energy. In LAMS youth, having a higher PGBI-10M score (≥12) at study entry was associated with high risk of developing BPSD,[21] as well as other severe psychopathology[2, 22] and disorders[15, 16] in the future.

The LAMS study also includes assessments of neuroimaging correlates of different trajectories of symptom dimensions in a subgroup (LAMS: n = 103; healthy peers: n = 40). Here, specific patterns of activity and functional connectivity in emotional regulation circuitry were associated with different pre-imaging PGBI-10M trajectories. Specifically, LAMS youth with initially high PGBI-10M trajectories showed decreased dorsolateral prefrontal cortical activity during a task measuring attentional control over emotional distracters, and decreased functional connectivity between ventrolateral prefrontal cortex and amygdala, relative to LAMS youth with lower PGBI-10M trajectories.[23] These findings indicate associations between patterns of functional abnormalities in emotional regulation circuitry and previous clinical course in youth with different psychiatric disorders.

The advance of surface-based methods for neuroimaging analyses has enabled measurement of regional thickness and surface area across the cerebral cortical mantle with submillimeter accuracy.[24–28] Interestingly, using dynamic mapping in 32 youth with emotional dysregulation disorders and/or behavioral developmental disorders, Gogtay et al. tracked gray matter cortical changes preceding the onset of their first manic episode.[29] A gradual increase over time in gray matter volume was shown in youth who converted to BPSD, relative to healthy control youth. Specifically, increased gray matter volume was shown in bilateral temporal cortices and in left ventrolateral prefrontal cortex in youth who converted to BPSD.[29] Global cortical thickness abnormalities were also reported in youth with ADHD in a longitudinal study of 163 youth with ADHD and 166 healthy controls.[30] Here, developmental trajectory analyses revealed a stable reduction over 5.7 years of left medial prefrontal cortical thickness in youth with ADHD who developed a worse clinical course than those with a better course (and controls).[31] Cortical abnormalities have also been reported in the superior temporal gyrus in youth with CD.[32] Together, these findings suggest that cortical thickness patterns may predispose to the development of specific psychiatric disorders in youth with emotional and/or behavioral developmental disorders. Yet, the extent to which patterns of cortical thickness can help distinguish youth with symptom trajectories, cutting across diagnoses,[33] remains unknown.

Machine learning is an area of artificial intelligence concerned with the development of algorithms and techniques able to automatically extract information from the data.[34] Recent evidence indicates that the combination of machine learning and neuroimaging techniques may help classify individuals, case by case, into different diagnostic groups or characterize individuals by different clinical course trajectories.[33]

In the present study, we aimed to use a combination of structural neuroimaging, specifically surface reconstruction, latent class analysis and machine learning to identify the extent to which patterns of cortical thickness could be used to accurately classify youth with different symptoms at the individual level. Specifically, we focused on trajectories of PGBI-10M scores, given the important relationships that we previously observed between PGBI-10M scores and development of future psychiatric disorders in LAMS youth (see above). *The absence of prior studies combining such approaches precluded formulation of specific hypotheses regarding the precise nature of cortical thickness patterns that would be the best classifiers of previous clinical course*. We thus examined 68 parcellated regions covering the cerebral cortical mantle, and aimed to determine the extent to which patterns of cortical thickness could differentiate youth with different clinical profiles. Specifically, this approach holds promise as a strategy to identify transdiagnostic pathophysiologic mechanisms of positive mood and energy dysregulation in youth with different emotional and/or behavioral developmental disorders.

## Method and materials

### Participants

The study received institutional review board approval at all scan sites (Case Western Reserve University [9–10–28], Cincinnati Children’s Hospital Medical Center [2010–3347], and University of Pittsburgh Medical Center [PRO10090442]). Parents or guardians provided written informed consent, and children provided written informed assent prior to study participation. Participants received monetary compensation and a framed picture of their structural neuroimaging scan. One-hundred-twenty-eight youth, recruited from the LAMS cohort of 685, and thirty-four newly recruited healthy comparison youth (HC) participated in the neuroimaging component of the LAMS study as follows: Case Western Reserve University (n = 32 LAMS; n = 14 HC); Cincinnati Children’s Hospital (n = 48 LAMS; 6 HC); and University of Pittsburgh Medical Center (n = 48 LAMS; 14 HC). All HC were recruited using local advertising at the three sites, and were free of any psychiatric disorder. Their first-degree relatives were free of mood disorders and psychosis, and their second-degree relatives were free of BPSD and any psychosis. Inclusion and exclusion criteria for LAMS youth have been previously described at length [22, 35] and synthesized in the S1 Materials and Table A in S1 Materials.

### Clinical assessment

Semi-annual parent/guardian’s assessments of PGBI-10M score,[20, 36] semi-annual parent and child assessments of anxiety symptoms using the Screen for Child Anxiety Related Emotional Disorders(SCARED),[37] and annual assessments of manic and depressive symptom severity using respectively the Schedule for Affective Disorders and Schizophrenia for School-Age Children(K-SADS) Mania Rating Scale (KMRS),[38] and Depression Rating Scale(KDRS),[39] were performed. Additionally, SCARED, KDRS, and KMRS were repeated on scan day. Exclusion criteria were: systemic medical illnesses, neurological disorders, history of trauma with loss of consciousness, use of central nervous system effecting medications, IQ<70 assessed by the Wechsler Abbreviated Scale of Intelligence (WASI), positive drug and/or alcohol screen on the day of MR scan, alcohol/substance abuse in the past 3 months (determined by the Schedule for Affective Disorders and Schizophrenia for School Age Children, Present and Lifetime Version; K-SADS-PL-W), significant visual disturbance, non-English speaker, history of physical/sexual abuse, autistic spectrum disorders/developmental delays, pregnancy, claustrophobia, and metal in the body.

### Neuroimaging protocol and surface reconstruction analysis

#### Structural acquisition

Data were acquired using an axial 3D MPRAGE sequence (TE/TI/TR = 3.29ms /900ms/2200ms, flip angle = 9, isotropic 1mm^3^ voxel, 192 axial slices, matrix size = 256x192; time: 7’02”).

#### Surface reconstruction analysis

**Fig 1:**
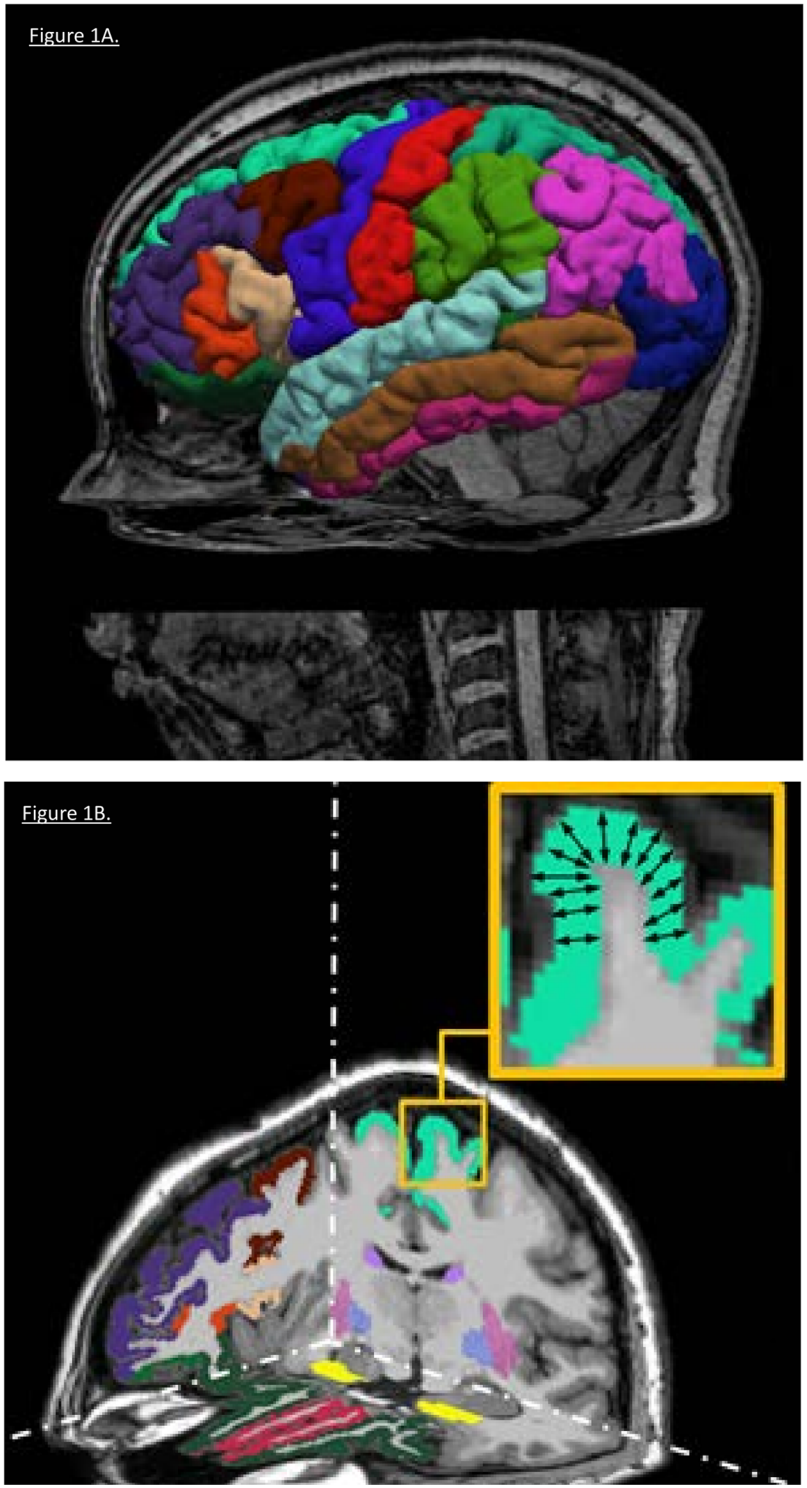
**Panel A**. Isosurface view of cortical surface reconstruction and cortical parcellation in one of our participants. **Panel B**. 3D view of cortical thickness of parcellated regions in the same participant. Here, few parcellated regions are displayed in native space, in accordance with the Freesurfer color-coding convention, and overimposed on the anatomical (mprage) image of the same participant.

Surface Reconstruction Analysis and quantification of thickness measurements were performed in 34 parcellated regions covering the cerebral cortical mantle in each hemisphere (Fig 1) using FreeSurfer software package (version-5.3; https://surfer.nmr.mgh.harvard.edu). This method has been extensively described in previous methodological papers [24–26] and is briefly described in the S1 Materials.

### Statistical analysis

#### Longitudinal trajectory analysis

Longitudinal Trajectory Analysis of PGBI-10M Scores was performed on the Statistical Analysis System (SAS) platform using *‘Proc Traj*’ software package, freely available at http://www.andrew.cmu.edu/user/bjones/index.html. *Proc Traj* is a specialized group-based mixture method that identifies distinct homogenous clusters (classes) of trajectories in a given population, over time.[40, 41]Trajectories of behaviors associated with difficulty regulating positive mood and energy were thereby determined in the 115 LAMS youth with usable neuroimaging data, using the semi-annual PGBI-10M ratings collected for up to 5 years. Previously employed to describe trajectories of depressive symptoms,[42, 43] the employment of *Proc Traj* in the present study allowed fitting two components simultaneously: 1. a censoring normal mixture model of the PGBI-10M score as a polynomial function of time, and 2. a latent class model using the multinomial logistic regression of the trajectory classification. The number of trajectories was determined based on (a) Bayesian Information Criterion, (b) posterior probabilities of group membership,[44] and (c) presence of a minimum of 10% of participants per trajectory. Trajectories were tested for linear, quadratic, and cubic trends. Z-scores, and associated p-values were then derived to determine the differences between slopes of main class-trajectories, using the following formula[45]:

$$\text{z=}\frac{\beta_{\;j}\;{- \beta }_{\;i}}{\sqrt{{\text{(SE}}_{\;\beta_{\;j}}{}^{\text{2}}{\text{+SE }}_{\beta_{\;i}}{}^{\text{2}})}}.$$

Between-class differences in demographic and clinical variables were examined using independent two-tailed t-tests or chi^2^ tests, as appropriate (Table B in the S1 Materials). Gender, age at scan, handedness, socioeconomic status (SES), site, signal to noise, BPSD diagnosis at scan (yes/no), depressive spectrum disorder diagnosis at scan (yes/no), ADHD diagnosis at scan (yes/no), and disruptive behavior disorder diagnosis (CD, ODD) at scan (yes/no) were entered as covariates of no-interest in this analysis. For a detailed description of how neuroimaging data were combined across site, see the S1 Materials.

#### Regularized linear logistic regression and machine learning analyses

Regularized Linear Logistic Regression and Machine Learning Analyses were performed on neuroimaging data after surface reconstruction analysis using *glmnet* and *caret* libraries, implemented in the R source package.[46] *Glmnet* is a freely available library (https://cran.r-project.org/web/packages/glmnet/index.html).[46] This includes fast algorithms for estimation of penalty terms (i.e., ℓ1 in least absolute shrinkage and selection operator (LASSO), ℓ2 in ridge regression and ℓ1- ℓ2 mixtures in elastic-net) for regularized linear regression models using cyclical coordinate descent, computed along a regularization path. Penalization techniques, such as ridge regression,[47] LASSO[48] and elastic-net[49] regression, have gained popularity over classical ordinary least squares estimates, allowing for the testing of a relatively large number of variables relative to the number of study participants, while minimizing the risk of inflating model error or overfitting. For example, ridge regression[47] minimizes the residual sum of squares, subject to a bound on the ℓ2-norm of the coefficients. As a continuous shrinkage method, ridge regression achieves its better prediction performance through a bias—variance trade-off, yet maintaining all the predictors in a given model. A more parsimonious alternative, namely the LASSO was proposed by Tibshirani.[48] By imposing an ℓ1-penalty on the regression coefficients, LASSO involves both continuous shrinkage and automatic variable selection, and simultaneously removes irrelevant predictors in a given model. While both techniques represent a valid approach in variable selection, LASSO is much more appealing due to its parsimoniousness. Yet, in the context of a dataset with highly inter-correlated variables, the selection of one variable over another is arbitrary. To overcome this limitation, a new regularization technique, namely elastic-net, has been recently proposed.[49] Elastic-net linearly combines the ℓ1 and ℓ2 penalty terms of ridge and LASSO methods. Thus, similar to ridge and LASSO, elastic-net simultaneously does automatic variable selection and continuous shrinkage, further adding a group-selection feature for highly correlated variables. All these techniques belong to the same family of regularized regression models where a scalar value (from 0 to 1) of the *alpha* parameter defines the weight of LASSO(ℓ1) versus ridge (ℓ2) optimization, where *alpha* = 1 represents LASSO regression, *alpha* close to 0 approaches ridge regression, and in-between *alpha* values represent elastic-net optimization. Thus, depending on the characteristics of a given dataset, one technique might be more appropriate than another. To identify the best model for our data, the Classification And REgression Training (*caret*; caret.r-forge.r-project.org) package was used. In *caret*, a set of functions relevant to machine learning, such as data splitting, pre-processing, feature selection, model tuning using resampling, variable importance estimation, and more, are available. In the present study, the combined use of *caret* and *glmnet* allowed for the identification of the optimal values of *alpha* (ridge vs elastic-net vs lasso) and *lambda* parameters. Elastic-net resulted to be the optimal model for our data (see results section). In brief, caret was used to: 1. stratify a random split of the data in training-test samples (60% and 40%, respectively using the createDataPartition function); 2. evaluate the effect of model tuning alpha and lambda parameters on performance (expand.grid function); 3. choose the “optimal” regularized regression model across these parameters from a training set (trainContol function; 10-fold cross-validation); and 4. estimate model performance in the training and test sample (summaryFunction, classProbs and predict functions). To this end, dROC library was used to provide measures of performance (sensitivity, specificity, accuracy; area under the curve; PPV and NPV) of this classification analysis. Specifically, ROC analysis provides tools to select optimal models and to discard suboptimal ones independently from (and prior to specifying) the cost context or the class distribution in diagnostic decision making.[50] Thus, 34 cortical thickness measures for each hemisphere (68 in total) were used as independent variables, with PGBI-10M trajectory classes, defined as described above, as dependent (outcome) variables. Gender, age at scan, handedness, IQ, SES, site, signal to noise ratio (see below), different diagnoses at scan including BPSD (yes/no), depressive spectrum disorder (yes/no), ADHD (yes/no), and disruptive behavior disorder (CD, ODD; yes/no), and 6 medication classes in the day of the scan, including mood stabilizer (ON/OFF), stimulant (ON/OFF), non-stimulant (ON/OFF), antidepressant (ON/OFF), antipsychotic (ON/OFF) and anxiolytic (ON/OFF) medications were entered as additional potential predictors in this analysis. Cortical thickness by age (or gender) interaction was also modelled in this analysis. The optimal regularized regression model was then used to derive the variable importance selection, based on the magnitude of the parameter estimates (i.e., betas). The sign (positive or negative) of the betas were used for interpretation the directionality of a given predictor (e.g., cortical thinning in a given brain region was derived by a negative beta).

#### Exploratory analyses

Two Multivariate Analyses of Variance (MANOVAs; one for patterns of cortical thickening and one for patterns of cortical thinning) were used to examine relationships between cortical thickness in brain regions that accurately distinguished the two extreme PGBI-10M trajectories and: KMRS, KDRS, SCARED scores in LAMS youth.

## Results

### Trajectory analysis

Due to data loss or failure to meet our data quality control criteria (see the S1 Materials), 13 participants were excluded from analyses (Table B in the S1 Materials), leaving 115 LAMS participants (13.6 y/o; M/F = 68/47;IQ = 100.1;R/L = 108/7) and 31 HC (12.5 y/o; M/F = 15/16;IQ = 104;R/L = 24/5). On the scan day, only one of the 115 LAMS youth did not have any diagnosis, 22 had a single diagnosis, and the other 92 had two or more comorbid diagnoses. 50.4% of LAMS youth were taking one or more medications on the scan date, including antidepressants, antipsychotics, mood stabilizers, stimulants, and/or non-stimulant ADHD medications. Demographic and clinical characteristics of the sample are reported in Table 1 (entire sample) and in Table B in the S1 Materials (main class-Trajectories).

**Table 1:** Demographic and clinical variable in 3 LAMS sites (Cleveland, Cincinnati and Pittsburgh).

	*SITE*	*N*	*Mean[SD]*	*Stats*.	*Sig*. *(2-sided)*
**Age at Scan**	CASE	44	13.3 [2.4]	F = 0.1	.934
	CINCI	45	13.3 [2.3]		
	PITT	57	13.5 [2.1]		
**Base IQ**	CASE	44	100.2 [17.8]	F = 0.5	.600
	CINCI	45	102.9 [13.6]		
	PITT	57	100.1 [15.2]		
**KMRS**	CASE	44	2.1 [4.8]	F = 2.6	.076
	CINCI	45	5.1 [7.4]		
	PITT	57	3.2 [6.2]		
**KDRS**	CASE	44	1.8 [4.1]	F = 2.7	.071
	CINCI	45	3.6 [4.0]		
	PITT	57	3.6 [4.7]		
**SCARED** ^*	CASE	44	9.2 [10.0]	F = 0.6	.527
	CINCI	40	11.2 [9.6]		
	PITT	57	11.4 [11.0]		
**Gender [M/F]**	CASE	44	0.5 [0.5]	F = 0.9	.413
	CINCI	45	0.4 [0.5]		
	PITT	57	0.4 [0.5]		
**SES [higher vs lower]** $	CASE	44	1.5 [0.5]	F = 1.0	.371
	CINCI	45	1.5 [0.5]		
	PITT	57	1.4 [0.5]		
**Handedness [L/R]**	CASE	44	1.1 [0.3]	F = 0.3	.747
	CINCI	43	1.1 [0.3]		
	PITT	57	1.1 [0.3]		
**PGBI-10M # (lower<12 / higher>13)**	CASE	26/5	—	χ2 = 0.8	.658
	CINCI	32/7	—		
	PITT	40/5	—		
**Bipolar Disorder at Scan [NO/YES]** #	CASE	18/13	—	χ2 = 4.0	.137
	CINCI	24/15	—		
	PITT	35/10	—		
**Depression at Scan [NO/YES]** #	CASE	30/1	—	χ2 = 1.8	.398
	CINCI	35/4	—		
	PITT	43/2	—		
**Anxiety at Scan [NO/YES]** #	CASE	30/1	—	χ2 = 6.4	0.041**
	CINCI	32/7	—		
	PITT	43/2	—		
**ADHD at Scan [NO/YES]** #	CASE	21/10	—	χ2 = 6.9	.140
	CINCI	19/20	—		
	PITT	18/26	—		
**Conduct-ODD-Disrupt at Scan [NO/YES]** #	CASE	28/3	—	χ2 = 13.5	0.001***
	CINCI	19/20	—		
	PITT	28/17	—		
**Substance Dependence at Scan [NO/YES]** #	CASE	30/1	—	χ2 = 2.7	.255
	CINCI	39/0	—		
	PITT	45/0	—		

^ Equal variances not assumed

* Missing info in 5 LAMS participants.

^#^ Data available in LAMS participants only

^$^ Lower SES includes No education, High School, GED, High School Diploma, Some Post-High School w/o degree or certification; Higher SES includes Associate's Degree, Other Post-High School certification, Bachelor's Degree or Higher

** Post-hoc analyses revealed that among LAMS youth included in this study, those recruited in the Cincinnati site had higher rate of Anxiety Disorders than the LAMS youth recruited from the Cleveland and Pittsburgh sites (p = .029 and p = 028, respectively).

*** Post-hoc analyses revealed that among LAMS youth included in this study, those recruited in the Cleveland site had lower rate of Conduct Disorder-Disruptive or Oppositional Defiant Disorders than the LAMS youth recruited from Cincinnati and Pittsburgh sites (p<0.001 and p = 009, respectively).

**Fig 2:**
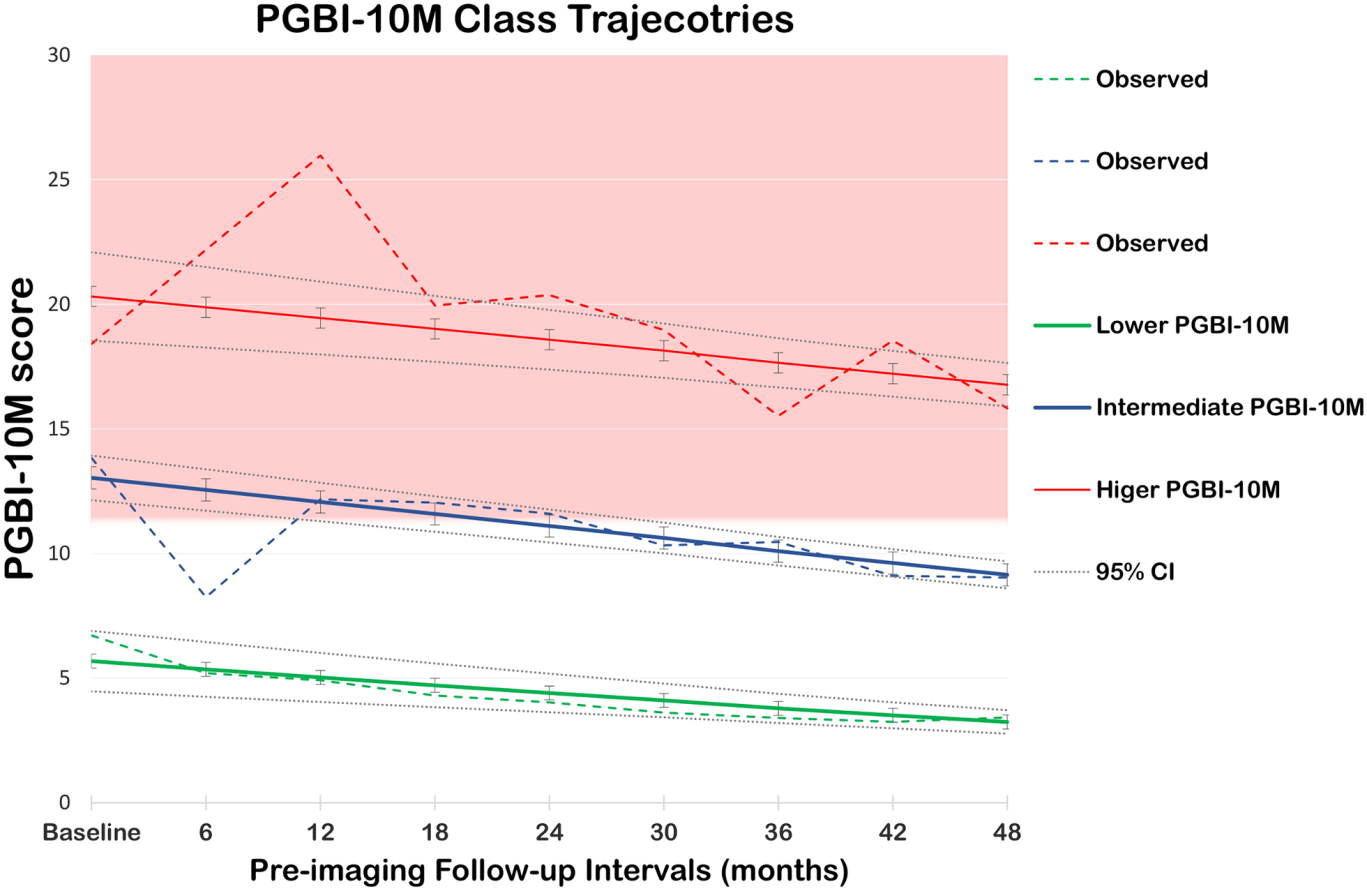
Line plot shows main class-trajectories identified in the 115 LAMS youth study participants. The red line represents the class-trajectory of LAMS youth with initially high and subsequently improving PGBI-10M scores, the blue line represents the class-trajectory of LAMS youth with intermediate PGBI-10 M scores and the green line represents the class-trajectory of LAMS youth with initially low and subsequently improving PGBI-10M scores in the pre-imaging follow-up period (5 years). The pink area represents the clinically significant range of PGBI-10M (>12).

Longitudinal Trajectory Analysis of PGBI-10M identified three (*1*. β0 = 16.2, β1 = -3.0; p = 0.05; *2*. β0 = 6.6, β1 = -5.0; p<0.001; *3*. β0 = 0.3, β1 = 0.3, β2 = 3.6; p = 0.07) main pre-imaging PGBI-10M class-trajectories in the 115 LAMS youth included in the study (Z_(class 1. vs class 2.)_ score = -3.24, p = 0.002; Z_(class 1. vs class 3.)_ score = 4.3, p<0.001; and Z_(class 2. vs class 3.)_ score = 4.8, p<0.001). Specifically, there were 18 youth (mean age[SD] = 14[2.2], gender ratio = 11/7 [F/M], handedness = 18/0, mean IQ[SD] = 98[17.3] SES = 33) with higher scores at study entry followed mostly by a stable course (i.e., PGBI-10M score ≥12 at all times in 6 out of 18 youth and in at least 50% of times in 17 out of 18 youth). Only 1 out of 18 youth showed an unstable course (i.e., PGBI-10M score <12 for more than 50% of the times); 61 youth (mean age[SD] = 13[2.1], gender ratio = 23/38 [F/M], handedness = 57/4, mean IQ[SD] = 97[14.7], and SES = 29) with ‘intermediate’ trajectories who were clinically more heterogeneous (i.e., 23 out of 61 youth showed PGBI-10M score ≥12 for at least 30% of the times); and 36 youth (mean age[SD] = 14[2.0], gender ratio = 12/24 [F/M], handedness = 33/3, mean IQ[SD] = 106[16.8], and SES = 34) with lower scores at study entry followed by a stable course (i.e., PGBI-10M score <12 at all times in 34 out of 36 youth. Two youth only reported a PGBI-10M score of 15 in one of the pre-imaging follow-up times) There were no main differences in total cerebral volume or total gray matter volume among the three principal PGBI-10M class-trajectories. (Fig 2 and Table 1).

### Elastic-net linear logistic regression and machine learning analyses

**Fig 3:**
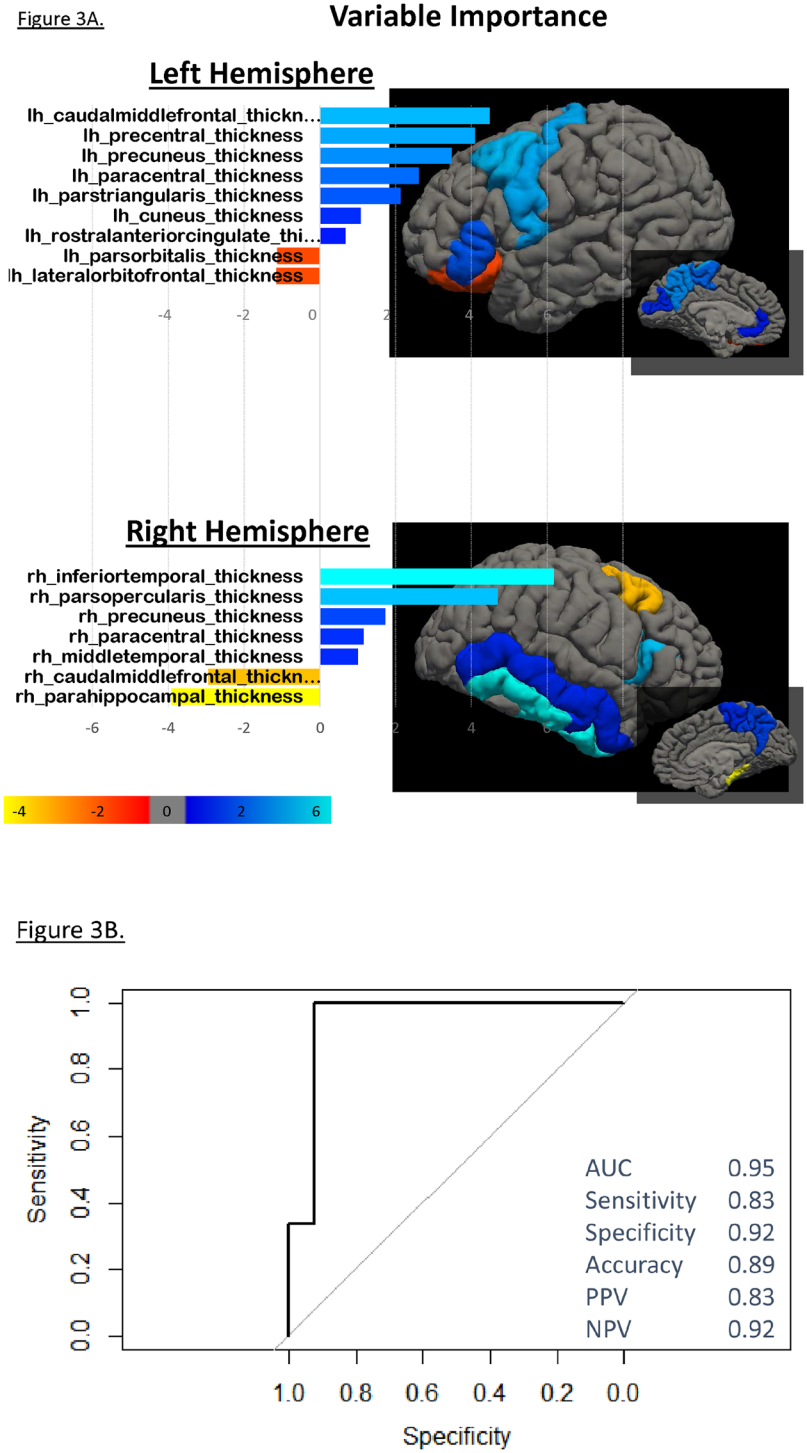
**Left Panel**. Bar plot represents cortical regions having from-higher-to-lower rank of importance in contributing to PGBI-10M trajectory-classification, i.e., differentiating LAMS youth with higher, from LAMS youth with lower, PGBI-10M trajectory. Blue bars represent regions in which greater cortical thickness contributed to this classification, while red bars represent regions in which lower cortical thickness contributed to this classification. Variable importance is also represented on the anatomical (mprage) image of one of our participant where color map reflects the relative contribution of each brain region using beta values, in accordance with the color-coding convention of the bar plot. Gray areas represent brain regions that did not contribute into the model. **Right Panel**. AUC plot.

Our main predictive analysis focused on the ability of cortical thickness measures to accurately classify LAMS youth into those with higher (n = 18) vs. those with lower (n = 36) pre-imaging PGBI-10M score trajectories. A training-test split (60%) of the sample was done and a 10-fold cross validation was used in the training phase to create this predictive model. Finally, predictors identified in the training phase were further validated on the test sample (40%) to estimate the accuracy of this model. Optimized parameters for the main predictive model (higher vs lower PGBI-10M trajectories) were *alpha* = 0.1, *lambda* = 0.1. *Alpha* values between 0 and 1 represent elastic-net optimization. Here, elastic-net linear logistic regression revealed that *patterns of cortical thickening* in key regions of the prefrontal cortex (i.e., left caudal middle frontal gyrus, pars triangularis and opercularis of the left inferior frontal gyrus), temporo-parietal cortex (right inferior and middle temporal gyrus, bilateral precuneus, and bilateral paracentral gyrus) and left cuneus, but *patterns of cortical thinning* in key regions of the prefrontal cortex (i.e., right caudal middle frontal gyrus, pars orbitalis of the left inferior frontal gyrus and left lateral orbital frontal gyrus) and the right parahippocampal gyrus of the temporal cortex accurately distinguished (statistics of this model upon the test sample: sensitivity = 0.83; specificity = 0.92; accuracy = 0.89; area under the curve = 0.95; Positive Predicted Value = 0.83 and Negative Predicted Value = 0.92; p = 0.03) youth with higher from those with lower PGBI-10M trajectories (Fig 3). Taking mood stabilizer medications and having a diagnosis of BPSD were also relevant predictors in distinguishing LAMS youth with higher, verus those with lower, pre-imaging PGBI-10M trajectories.

Two additional analyses determined the extent to which cortical thickness could accurately classify LAMS youth with higher PGB-10M trajectories vs. control youth (n = 31) and LAMS youth with lower PGB-10M trajectories vs. control youth (see additional results, Figure A and Figure B in the S1 Materials).

### Exploratory analyses

In the 18 LAMS youth with higher PGBI-10M trajectories and the 36 LAMS youth with lower PGBI-10M trajectories, there were no significant relationships among clinical variables, including KMRS, KDRS, SCARED scores on the day of the scan and cortical thickness in any of the regions that discriminated the two extreme PGBI-10M trajectories (for patterns of cortical thickening, see Table C in the S1 Materials; for patterns of cortical thinning, see Table D in the S1 Materials).

## Discussion

Our main findings revealed that patterns of widespread cortical thickening in portions of the left dorsolateral prefrontal cortex (including the caudal middle frontal gyrus and pars triangularis of the inferior frontal gyrus), medial parietal (left and right precuneus) cortex and lateral portions of the right temporal cortex, but cortical thinning in the right dorsolateral prefrontal cortex(including the caudal middle frontal gyrus), left ventrolateral prefrontal cortex (including the lateral orbitofrontal gyrus and the pars orbitalis of the inferior frontal gyrus) and right parahippocampal gyrus accurately distinguished LAMS youth with higher, from those with lower, PGBI-10M trajectories.

These findings parallel previous findings of predominantly increased cortical volumes in a sample of 32 youth with emotional dysregulation and/or behavioral disorders who converted to BPSD (relative to healthy youth).[29] Importantly, in the present study we did not focus on single diagnoses, but rather looked at different trajectories of positive mood and energy dysregulation up to five years that cut across diagnoses. This might in part explain the differences in findings of the present study with those observed in Gogtay et al. Decreases in frontal cortical thickness in adolescents with BPSD relative to healthy control youth were also reported,[51] but only generic measures of cortical thickness for frontal, temporal and parietal lobes were provided in this latter study. Thus, further speculations are not possible. Recent studies reported increased prefrontal cortical thickness (inferior frontal gyrus) in youth at high genetic risk of mood disorders who subsequently developed depressive symptoms.[52] In these youth, reduced cortical thickness in the right parahippocampal(50) (and right fusiform gyri)[52, 53] were also reported. Together, these findings indicate that combinations of increased and decreased cortical thickness are associated with presence of, and/or risk for future, psychiatric disorders, and partially parallel findings in the present study.

There are also some remarkable parallels between findings from the present study and our previous LAMS neuroimaging studies. For example, we previously showed a positive correlation between PGBI-10M-score at scan and left middle prefrontal cortical activity to win trials during a reward paradigm in LAMS youth.[54] Our present finding of greater cortical thickness in the left caudal middle frontal gyrus in LAMS youth with higher PGBI-10M trajectories than in LAMS youth with lower PGBI-10M trajectories parallel this finding, and suggest that cortical abnormalities in the left middle frontal gyrus may underline heightened reward sensitivity and greater attention to reward stimuli in emotionally and behaviorally dysregulated youth, regardless of diagnosis.

There are limitations. 1. Specifically, trajectories were based on pre-imaging clinical assessments and only one neuroimaging scan was acquired. Future studies with post-imaging clinical trajectories and more than one neuroimaging measure are needed to determine the relationship between developmental changes in neuroimaging measures and how these changes can predict future symptom trajectories. 2. In this study, we focused on the two extreme (higher and lower) PGBI-10M classes. These two classes are clinically distinct, and therefore appropriate for the identification of a discriminant algorithm. It is worth mentioning that these classes resemble the two main PGBI-10M classes that were previously reported in the entire LAMS sample (n = 707) using 24-month trajectories (see Table E in S1 Materials).[23, 35] To take advantage of the unique longitudinal design, in the present study we focused on the subsample of LAMS youth with neuroimaging data who were clinically characterized for up to 5 years. This is the only study that combines such a long longitudinal design and neuroimaging. The number of youth within these classes is, however, relatively small and this represents a limitation of the study. Yet, the Positive and Negative Predicted Values (PPV = 0.83 and NPV = 0.92, respectively) were high and indicated that the neuroimaging patterns identified in the training sample were able to accurately classify 5 out of 6 youth with higher (PPV) and 12 out of 13 youth with lower (NPV) PGBI-10M trajectories in the independent (i.e., randomly selected a priori) testing sample. 3. The 5-year trajectories were based on the PGBI-10M, which is a parental report of the child’s difficulty regulating positive mood and energy. While parental reports are largely employed in research settings as a way to collect clinically relevant information based on mile-stone assessments of observed behaviors (i.e., the observation of the child’s ‘performance’ in a natural setting), these instruments might lack of norm-reference (i.e., limitation of a parent in comparing the functioning of his own child in relation to other children). 4. Cortical subregions were parcellated using FreeSurfer, which offers a fully automated parcellation method. Parcellated regions were then visually inspected for gross artifacts. In addition, a data quality protocol was used, in accordance with the protocol proposed by the ENIGMA project (http://enigma.ini.usc.edu/protocols/imaging-protocols; see the S1 Materials). While a recent study comparing measures of manually edited vs. unedited FreeSurfer cortical regions did not find significant between-method differences,[55] inaccuracies can occur in automated parcellation methods and further studies may be needed to quantify the accuracy of these measures.

Importantly, the present study indicates for the first time how the combined use of cortical thickness measures, symptom trajectory analysis and machine learning can identify clusters of neural regions that accurately classify individual youth into groups defined by symptom trajectories over time, specifically trajectories of observed behaviors associated with positive mood and energy regulation, in a large sample of youth recruited transdiagnostically. This approach holds promise as a strategy to identify objective neural markers reflecting pathophysiologic mechanisms underlying emotional dysregulation that can classify individual youth, case by case, in terms of clinical course. Future studies can use such an approach to identify patterns of neural markers that predict future clinical course.

## Supporting information

S1 Materials Additional information concerning neuroimaging acquisition and quality control procedure in neuroimaging data analyses, additional results in LAMS Youth with Higher (or Lower) PGBI-10M Trajectories vs typically developing youth, supplemental tables (i.e., Table A-D) and figures (i.e., Figure A-B). (DOCX)
