# Supplementary material for: Using machine learning and surface reconstruction to accurately differentiate different trajectories of mood and energy dysregulation in youth
Source: PLoS One. 2017 Jul 6;12(7):e0180221. doi: 10.1371/journal.pone.0180221 (PMC5500381; doi:10.1371/journal.pone.0180221)
Supplement: S1 Materials — (DOCX) [file pone.0180221.s001.docx]

**S1 Methods:**

**Neuroimaging Acquisition**. Structural images were corrected for non-uniformity of intensity, registered to Talairach space with an affine transformation, normalized for intensity again and automatically skull stripped and segmented. Segmented and skull stripped data of all participants were visually inspected and gross inaccuracies were manually corrected by an expert neuroanatomist (A.V.), to avoid misclassification of tissue type. Finally, maps were smoothed by a Gaussian Kernel of 10-mm Full-Width at Half Maximum (FWHM) and mean cortical thickness values were calculated for 34 brain regions (Desikan parcellation), for both hemispheres and for hemispheres themselves, for each participant. Cortical thickness at each point was calculated as the average of two values, the minimal distance from gray/white matter boundary and pial surface and vice versa.[[16]](http://onlinelibrary.wiley.com/doi/10.1111/jon.12259/full#jon12259-bib-0016) This method uses intensity and continuity information from the whole 3D volume, thus cortical maps are not simply based on absolute signal intensity but on intensity gradients across brain tissues. Technique accuracy has been validated by direct comparisons with manual measures on both MRI and post-mortem data.[[17]](http://onlinelibrary.wiley.com/doi/10.1111/jon.12259/full#jon12259-bib-0017) Each brain's subject was morphed and registered to an average spherical surface that finely aligns sulci and gyri across them, so that cortical thickness values can be mapped on this average inflated surface, avoiding interference of cortical folding on the visualization.

**Quality Control Procedure.** In accordance with the quality control procedure proposed by the ENIGMA project (<http://enigma.ini.usc.edu/protocols/imaging-protocols>) for structural images processed in Freesurfer (or FSL), neuroimaging data was visually inspected to identify gross anatomical and/or imaging-related artifacts. In addition, three steps have been used: 1. Outlier Detection: An R script identified participants with cortical thickness values that deviated from the majority of study participants (>2SD from the mean). Given that being an outlier does not necessarily mean that the dataset should be excluded from the analysis, AV inspected these datasets closely to ensure the data identified in step 1 were processed properly in Freesurfer. Thus, if the brain of an outlier had parcellated properly (step1, step2 and step3), this dataset has been included in the analyses. 2. Internal Surface Method: A Matlab function was used to plot cortical surface segmentations directly on to a participant’s scan and collate snapshots of orthogonal views of the segmented brain into a webpage for checking. 3. External Surface Method: A webpage with medial and lateral views of the surface segmentation was produced for easy checking, using Freeview.

**Combining Data across Sites.** To control for inter-site scanner variability and to combine neuroimaging data across the three sites, all the potential predictive variables were normalized. In addition, standards by the Biomedical Informatics Research Network (BIRN; <http://www.nbirn.net>) for data acquisition and information sharing were implemented. Using an f-BIRN phantom and recommended procedures, scanner signal-to-noise-ratio (SNR) was collected and monitored for stability on a monthly basis at each scanner site,[[1](#_ENREF_1), [2](#_ENREF_2)] and estimates of SNR were entered as a covariate in all analyses. Moreover, scanning site was also entered as a covariate in all analyses (site effect on demographic and clinical variables is reported in Table1).

**S1 Results:**

**Elastic-net Linear Logistic Regression and Machine Learning Analyses in LAMS Youth with Higher PGBI-10M Trajectories (n=18) vs typically developing youth (n=31).**

Paralleling the main analyses age, gender, headedness, IQ, SNR, and the cortical thickness measures of the 68 parcellated anatomical regions were entered in the model as possible predictors. Optimized parameters for this predictive model (high PGBI-10M trajectory vs controls) were *alpha*=0.4 and *lambda*=0.1. Thus, elastic-net linear logistic regression revealed that higher cortical thickening in the prefrontal cortex (i.e., right rostral middle frontal gyrus), in the parietal cortex (left inferior parietal lobule, bilateral superior parietal lobules and paracentral gyrus), but cortical thinning in the entorhinal cortex (bilaterally) and right insula predicted the membership of youth with higher PGBI-10M trajectories, when compared to age, gender, SES-matched typically developing youth (sensitivity=0.83; specificity=1; accuracy=0.94; area under the curve=0.9; PPV=15 and NPV=1 in an N=16 test sample; eFigure1A).

**LASSO Linear Logistic Regression and Machine Learning Analyses in LAMS Youth with Lower PGBI-10M Trajectories (n=36) vs typically developing youth (n=31).**

Similarly, optimized parameters for this predictive model (lower PGBI-10M trajectory vs controls) were *alpha*=1 and *lambda*=0.1. Thus, LASSO linear logistic regression revealed that higher cortical thickness in the temporo-parietal cortex (i.e., left fusiform gyrus, right entorhinal gyrus, right inferior parietal lobule and right paracentral gyrus), but cortical thinning in the frontal cortex (i.e., right rostral middle frontal gyrus); parietal cortex (i.e., left cuneus and superior parietal lobule), and occipital cortex (i.e., right lingual cortex) predicted the membership of youth with lower PGBI-10M trajectories, when compared to age, gender, IQ, SES-matched typically developing youth. (sensitivity=0.86; specificity=1; accuracy=0.93; AUC=1; PPV=12 and NPV=1 in an N=13 test sample; eFigure1B).

**Table A.**

**Demographic and Clinical Variables in included and excluded participants.**

|  | *EXCLUDED* | |  | *INCLUDED* | |  | *BETWEEN-GROUP* | |
| --- | --- | --- | --- | --- | --- | --- | --- | --- |
|  |  |  |  |  |  |  |  |  |
|  | *N* | *Mean[SD]* |  | *N* | *Mean[SD]* |  | *Stats.* | *Sig. (2-sided)* |
|  |  |  |  |  |  |  |  |  |
| Age At Scan^ | 13 | 13.5 [1.3] |  | 146 | 13.4 [2.2] |  | t = 0.4 | .688 |
| Base IQ | 13 | 111.9 [15.0] |  | 146 | 101.0 [15.5] |  | t =2.4 | **.020** |
| KMRS | 13 | 3.7 [6.4] |  | 146 | 3.5 [6.3] |  | t =0.1 | .910 |
| KDRS | 13 | 2.3 [3.4] |  | 146 | 3.1 [4.4] |  | t =-0.6 | .576 |
| SCARED * | 12 | 8.9 [10.2] |  | 141 | 10.7 [10.3] |  | t =-0.6 | .577 |
| Gender [M/F] | 10/3 | -- |  | 83/63 | -- |  | χ2 =1.9 | .159 |
| SES ^$^ | 0/1/2/8/2 | -- |  | 8/33/37/47/34 | -- |  | χ2 =7.4 | .117 |
| Handedness [L/R] | 11/2 | -- |  | 132/14 | -- |  | χ2 =0.4 | .506 |
| PGBI-10M ^#^ (lower<12 / higher>13) | 10/3 | -- |  | 98/17 | -- |  | χ2 =0.6 | .435 |
| Bipolar Disorder at Scan [NO/YES] ^#^ | 6/7 | -- |  | 77/38 | -- |  | χ2 =2.2 | .136 |
| Depression at Scan [NO/YES] ^#^ | 12/1 | -- |  | 108/7 | -- |  | χ2 =0.5 | .821 |
| Anxiety at Scan [NO/YES] ^#^ | 13/0 | -- |  | 105/10 | -- |  | χ2 =1.2 | .268 |
| ADHD at Scan [NO/YES] ^#^ | 6/7 | -- |  | 58/57 | -- |  | χ2 =0.8 | .770 |
| Conduct-ODD-Disrupt at Scan [NO/YES] ^#^ | 10/3 | -- |  | 75/40 | -- |  | χ2 =0.7 | .397 |

*^ Equal variances not assumed.*

** Missing info in 6 participants of whom one was excluded and 5 were included in the study.*

*# Data available in LAMS participants only*

*$ Level 1. No education or High School, Level 2. GED or High School Diploma, Level 3. Some Post-High School w/o degree or certification; Level 4. Higher SES includes Associate's Degree or Other Post-High School certification, Level 5. Bachelor's Degree or Higher*

**Table B.**

**Demographic and Clinical Variables in 3 main PBGI-10M class-trajectories of LAMS Youth and Healthy Youth.**

|  | Class-Trajectories | N | Mean[SD] | STATS | Sig. (2-sided) | |
| --- | --- | --- | --- | --- | --- | --- |
|  |  |  |  |  | |  |
|  |  |  |  |  | |  |
| Age At Scan | LOW | 36 | 14[2.0] | F[2,114]= 0.9 | | .388 |
|  | INTERMEDIATE | 61 | 13[2.1] |  | |  |
|  | HIGH | 18 | 14[2.2] |  | |  |
| **Base IQ** | LOW | 36 | 106[16.8] | **F[2,114]= 3.8** | | **.024** |
|  | INTERMEDIATE | 61 | 97[14.6] |  | |  |
|  | HIGH | 18 | 98[17.5] |  | |  |
| SES [higher vs lower] $ | LOW | 36 | 35[1.1] | F[2,114]= 0.9 | | .060 |
|  | INTERMEDIATE | 61 | 29[1.3] |  | |  |
|  | HIGH | 18 | 33[1.1] |  | |  |
| KDRS | LOW | 36 | 2.6 [3.5] | F[2,114]= 3.0 | | 0.051 |
|  | INTERMEDIATE | 61 | 4.0 [4.6] |  | |  |
|  | HIGH | 18 | 6.2 [6.4] |  | |  |
| **KMRS** | LOW | 36 | 1.8 [4.3] | **F[2,114]= 6.0** | | **0.003***** |
|  | INTERMEDIATE | 61 | 4.0 [5.6] |  | |  |
|  | HIGH | 18 | 8.6 [9.3] |  | |  |
| SCARED ^* | LOW | 31 | 8.2 [7.6] | F[2,114]= 2.3 | | .104 |
|  | INTERMEDIATE | 59 | 13.1 [11.3] |  | |  |
|  | HIGH | 18 | 14.3 [13.2] |  | |  |
| Gender [M/F] | LOW | 22/14 |  | c2 =4.1 | | .126 |
|  | INTERMEDIATE | 39/22 |  |  | |  |
|  | HIGH | 7/11 |  |  | |  |
| Handedness [L/R] | LOW | 35/1 |  | c2 =1.5 | | .470 |
|  | INTERMEDIATE | 55/6 |  |  | |  |
|  | HIGH | 18/0 |  |  | |  |
| **Bipolar Disorder at Scan [NO/YES] #** | LOW | 33/3 |  | **c2 =22.7** | | **<.001** |
|  | INTERMEDIATE | 40/22 |  |  | |  |
|  | HIGH | 5/13 |  |  | |  |
| Depression at Scan [NO/YES] # | LOW | 34/2 |  | c2 =.3 | | .857 |
|  | INTERMEDIATE | 56/4 |  |  | |  |
|  | HIGH | 17/1 |  |  | |  |
| Anxiety at Scan [NO/YES] # | LOW | 33/3 |  | c2 =1.7 | | .407 |
|  | INTERMEDIATE | 57/4 |  |  | |  |
|  | HIGH | 15/3 |  |  | |  |
| **ADHD at Scan [NO/YES] #** | LOW | 26/10 |  | **c2 =9.2** | | **.010** |
|  | INTERMEDIATE | 25/36 |  |  | |  |
|  | HIGH | 8/10 |  |  | |  |
| **Conduct-ODD-Disrupt at Scan [NO/YES] #** | LOW | 28/8 |  | **c2 =5.9** | | **.050** |
|  | INTERMEDIATE | 39/22 |  |  | |  |
|  | HIGH | 8/10 |  |  | |  |
| Substance Dependence at Scan [NO/YES] # | LOW | 36/0 |  | c2 =0.9 | | .640 |
|  | INTERMEDIATE | 60/1 |  |  | |  |
|  | HIGH | 18/0 |  |  | |  |

*^ Equal variances not assumed*

** Missing info in 5 LAMS participants.*

*# Data avaiable in LAMS participants only*

*$ Lower SES includes No education, High School, GED, High School Diploma, Some Post-High School w/o degree or certiffication; Higher SES includes Associate's Degree, Other Post-High School certification, Bachelor's Degree or Higher.*

*** Post-hoc analyses revealed that LAMS youth with low PBGI-10M were older than the Healthy Controls (p=0.005).*

**** Post-hoc analyses revealed that Healthy Control Youth reported lower manic symptoms than the LAMS youth with low (p=0.003), intermedate (p=0.015) and high (<0.0001) PBGI-10M trajectories.*

***** Post-hoc analyses revealed that Healthy Control Youth reported lower depressive and anxiety symptoms than the LAMS youth with intermedate (p<0.001) and high (<0.001 and p=0.003, respectively) PBGI-10M trajectories.*

**Table C.**

**Multivariate Analysis of Variance to examine a main effect of clinical symptom severity (KMRS, KDRS, SCARED) at the time of the scan on the patter of brain regions showing cortical thickening distinguishing LAMS youth with higher, from those with lower, PGBI-10M trajectories.**

| **A.** **Regions with cortical thickening** |  | ***Wilks' Lambda Value*** | ***F*** | ***Sig.*** |
| --- | --- | --- | --- | --- |
| KMRS at scan | |  | 0.7 | 1.4 |
| KDRS at scan | |  | 0.8 | 1.2 |
| Scared at scan |  | 0.9 | 0.5 | 0.78 |
|  |  |  |  |  |
|  | |  |  |  |

*Brain regions showing cortical thickening in LAMS youth with higher PGBI-10M trajectories were the caudal middle frontal gyrus (bilaterally), left pars triangularis of the inferior frontal gyrus, left rostral anterior cingulate gyrus, right parahippocampal gyrus, right middle temporal gyrus, precuneus (bilaterally), and left paracentral gyrus and left cuneus.*

**Table D.**

**Multivariate Analysis of Variance to examine a main effect of clinical symptom severity (KMRS, KDRS, SCARED) at the time of the scan on the patter of brain regions showing cortical thinning distinguishing LAMS youth with higher, from those with lower, PGBI-10M trajectories.**

| **B. Regions with cortical thinning** |  | ***Wilks' Lambda Value*** | ***F*** | ***Sig.*** |
| --- | --- | --- | --- | --- |
| KMRS at scan |  | 0.9 | 1.1 | .382 |
| KDRS at scan |  | 1.0 | 0.6 | .724 |
| SCARED at scan |  | 1.0 | 0.6 | .794 |

*Brain regions showing a cortical thinning in LAMS youth with higher PGBI-10M trajectories were the pars opercularis of the right inferior frontal gyrus, left lateral orbital frontal gyrus, right inferior temporal gyrus and right inferior parietal gyrus.*

*KMRD= Schedule for Affective Disorders and Schizophrenia for School-Age Children Mania Rating Scale;[*[*3*](#_ENREF_3)*] KDRS= Schedule for Affective Disorders and Schizophrenia for School-Age Children Depression Rating Scale;[*[*4*](#_ENREF_4)*] SCARED= Screen for Child Anxiety Related Emotional Disorders.[*[*5*](#_ENREF_5)*]*

**Table E.**

**Two-way cross table between the 5-year class trajectories identified in the present study and the 24-month trajectories identified in Findling et al. 2013.[**[**6**](#_ENREF_6)**]**

|  | | *24-MONTH*  *TRAJECTORIES* | | | |  |
| --- | --- | --- | --- | --- | --- | --- |
|  |  | **High & rising** | **High & reducing** | **Unstable** | **Low & reducing** | Total |
| *5-YEAR TRAJECTORIES* | **Higher PGBI-10M** | 13 | 4 | 1 | 0 | **18** |
|  | **Intermed. PGBI-10M** | 0 | 36 | 4 | 21 | **61** |
|  | **Lower PGBI-10M** |  | 0 | 0 | 36 | **36** |
|  | Total | 13 | 40 | 5 | 57 | **115** |

*The association between the two ordinal variables was highly significant (Somers' d=0.8, T=12.9, p<0.001).*

**Figure A.**

**Bar plot represents cortical regions having from-higher-to-lower rank of importance in contributing to the accurate classification of LAMS youth with lower PGBI-10M trajectories and healthy controls. Blue bars represent thicker anatomical cortical regions, while red bars represent thinner anatomical cortical regions.**


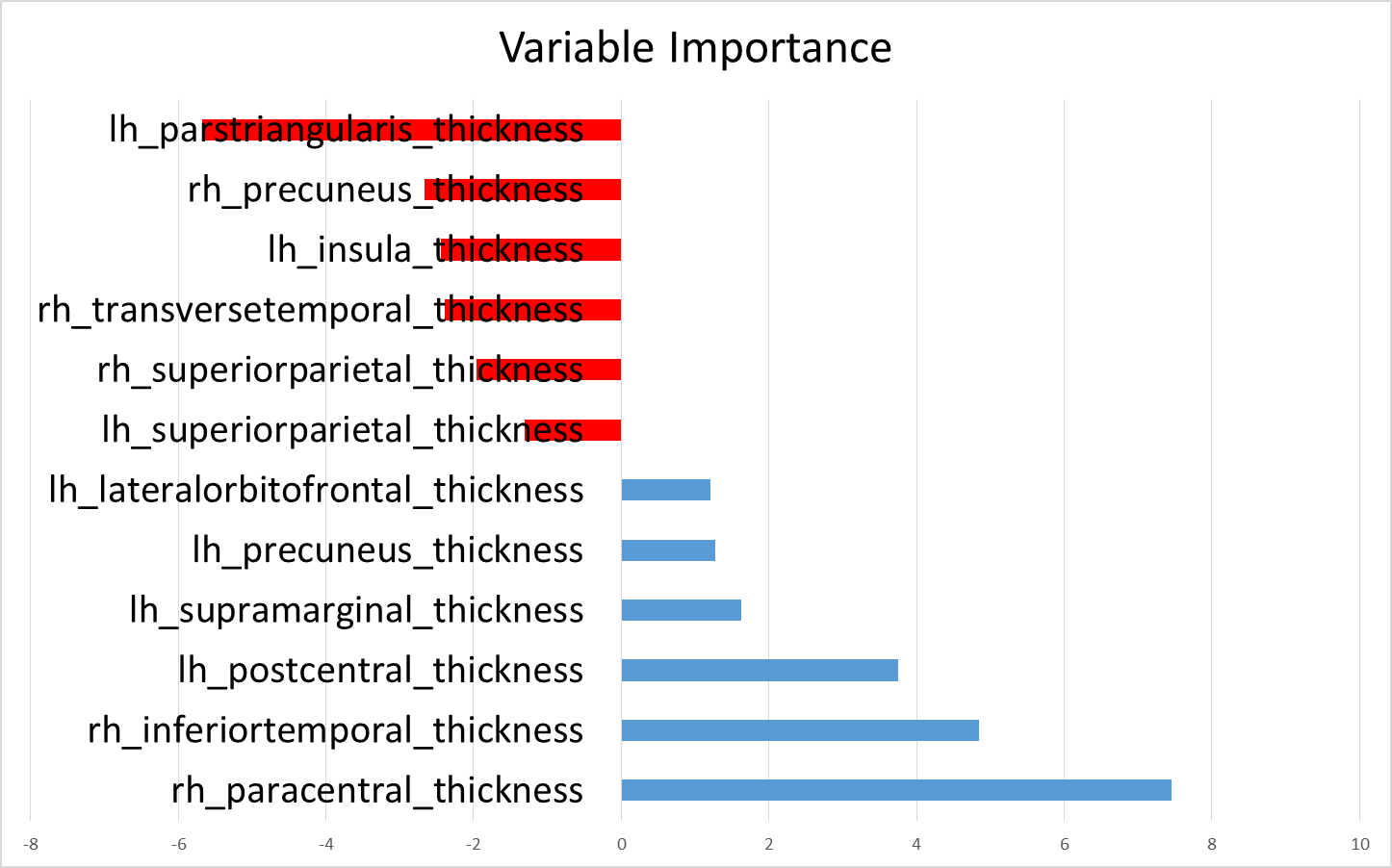


**Figure B.**

**Bar plot represents cortical regions having from-higher-to-lower rank of importance in contributing to the accurate classification of LAMS youth with higher PGBI-10M trajectories and healthy controls.. Blue bars represent thicker anatomical cortical regions, while red bars represent thinner anatomical cortical regions.**

**S1 References**

1. Friedman L, Glover GH. Report on a multicenter fMRI quality assurance protocol. J Magn Reson Imaging. 2006;23(6):827-39. Epub 2006/05/02. doi: 10.1002/jmri.20583. PubMed PMID: 16649196.

2. Friedman L, Glover GH. Reducing interscanner variability of activation in a multicenter fMRI study: controlling for signal-to-fluctuation-noise-ratio (SFNR) differences. Neuroimage. 2006;33(2):471-81.

3. Axelson D, Birmaher BJ, Brent D, Wassick S, Hoover C, Bridge J, et al. A preliminary study of the Kiddie Schedule for Affective Disorders and Schizophrenia for School-Age Children mania rating scale for children and adolescents. J Child Adolesc Psychopharmacol. 2003;13(4):463-70. Epub 2004/02/24. doi: 10.1089/104454603322724850. PubMed PMID: 14977459.

4. Kaufman J, Birmaher B, Brent D, Rao UMA, Flynn C, Moreci P, et al. Schedule for Affective Disorders and Schizophrenia for School-Age Children-Present and Lifetime Version (K-SADS-PL): Initial Reliability and Validity Data. Journal of the American Academy of Child and Adolescent Psychiatry. 1997;36(7):980-8. doi: Doi: 10.1097/00004583-199707000-00021.

5. Birmaher B, Khetarpal S, Brent D, Cully M, Balach L, Kaufman J, et al. The Screen for Child Anxiety Related Emotional Disorders (SCARED): scale construction and psychometric characteristics. Journal of the American Academy of Child and Adolescent Psychiatry. 1997;36(4):545-53. Epub 1997/04/01. doi: S0890-8567(09)62526-0 [pii]

10.1097/00004583-199704000-00018. PubMed PMID: 9100430.

6. Findling RL, Jo B, Frazier TW, Youngstrom EA, Demeter CA, Fristad MA, et al. The 24-month course of manic symptoms in children. Bipolar Disord. 2013;15(6):669-79. Epub 2013/06/27. doi: 10.1111/bdi.12100. PubMed PMID: 23799945; PubMed Central PMCID: PMC3762908.
